# Indents

**Published:** 1923-03

**Authors:** 


### INDENTS

**☞ Brief Articles of Technical or Scientific Value will receive notice and comment. Contributions invited.**

---

THE CUSPIDS AS "PROTECTORS OF THE INCISORS." The artificial cuspids might be termed "The Protectors of the Incisors," for certainly such protection during biting and mastication of food is one of their chief functions, and when they are rightly placed, they achieve it so admirably that the dentist may give his patient the advantage of the very longest bite artificial teeth (with their great biting efficiency and facility of clear speech) and never have a broken tooth. This is the experience of hundreds of dentists who properly articulate the cuspids.

When the cuspids are not set to proper relations, one of two things happens: either the upper cuspids are given an unnatural and undesirable appearance, or the strain in lateral articulation is brought upon the incisors and they are broken from the plate, by the form of contact illustrated above.

When the lower jaw is at rest in central occlusion, the lower cuspid should occlude with the distal portion of the upper lateral and the mesial facet of the upper cuspid. When the jaw moves to one side, with the teeth in contact, the lower cuspid slides easily in contact with both these upper teeth, without bringing pressure on the lateral.

---

ARTICULATING THE CUSPIDS. Correct cuspid relations are accomplished by the following steps:

Set the upper and lower cuspids.

Grind the cutting edge of the lower jaw cuspid until the tip of the cusp passes smoothly and evenly through the interdental space between the upper lateral and cuspid.

Grind the edge of the upper cuspid until its tip passes just through the space between the lower cuspid and bicuspid. *It should not "ride" either tooth.* This is very important.

Incline the facets on the cutting edges of both teeth very flatly, one downward and outward and one upward and inward.

Do not set the lower cuspid to a deep underbite.

Wax the teeth in position and move the articulator in articulation. The lower cuspids should slide in contact with the upper laterals, but one tooth should not dislodge the other from its position even when lightly waxed. *The importance of this can not be over-emphasized.*

When the plates are vulcanized and in the mouth, this relation should receive careful attention, and the teeth should be ground until it can be clearly seen that it has been established.

Correct relations of upper and lower cuspids can not be secured on plain line articulators. The best that can be done, if such articulators are used, is to try to secure them in the mouth after the dentures are otherwise finished. This is much less satisfactory in every way than obtaining them by the use of an anatomical articulator and making only final adjustments in the mouth.

Correct relations of upper and lower cuspids can be much better obtained with Trubyte teeth than with others because they are shaped with a knowledge of the engineering necessities of artificial denture articulation. When dentists have mastered the principle of correct cuspid positions, very slight changes in Trubyte cuspid forms are necessary.

When Trubyte cuspids are correctly placed, they take up, with the bicuspids and molars, the force of closure of the jaws and *protect the incisors from improper and destructive contacts.*

The lower incisors may be set to proper occluding and articulating relations. They must not so deeply underbite the upper incisors that they will not "clear" in lateral movements of the articulator.

They should be set so as to leave a space between them and their antagonists when the lower jaw is in center occlusion.

The lower lateral and central should have a sliding contact with the upper antagonizing incisors on the side of mouth on which working contact of posteriors is being made.

---

THE PLACE OF GRINDING IN DENTURE MAKING. No artificial dentures can be regarded as finished which are not properly ground.

Some dentists think that if artificial teeth are properly formed, they should require no grinding. *This idea is mistaken.* Trubyte teeth have been carved to articulate with the greatest possible precision and can be set to very fine articulation without any grinding. But this is not desirable from the patient's viewpoint.

Teeth need to be ground to remove little points which prevent close adaptation, to form facets which are capable of crushing food elements, to give those facets sharp edges that they may cut up fibers, to remove the glaze from the facets to increase their efficiency, and *to make sure that the facets are formed in harmony with the habitual jaw movements of the patient,* which is essential to their greatest efficiency.

The necessity for this form and extent of grinding should not be held to excuse the use of teeth which are not properly formed by the manufacturer. Unless teeth have been carefully and scientifically prepared for such grinding, it can not be done by the dentist. Trubyte teeth are immeasurably better prepared for such grinding than any other artificial teeth.

Such grinding can not usually be accomplished with a stone, save perhaps for a spot here and there or for the shaping of some small areas, such as the edges of the cuspids. Extensive grinding with a stone is practically certain to destroy the masticating efficiency of the teeth.

Trubyte teeth present high cusps, deep fossæ and sulci, *shallow bite* and many points of contact with opposing teeth. They may be ground to the highest efficiency possible to artificial teeth by the simple method described on the following page.

---

GRINDING BY THE PATIENT. When the dentures are vulcanized and in the mouth, they should be examined for any points of contact with undue pressure, and such points should be relieved. The finest adaptation can be secured by smearing the incisal and occlusal surfaces of the teeth with a mixture of cocoa butter and carborundum and causing the patient to grind the dentures to final adaptation. We have never had a patient who was not desirous of doing this, when the benefits were explained.

When the patient commences the "side to side movement"—in grinding to place, note carefully whether the grinding causes either denture to rotate. Any rotation is probably caused by the interference of cusp planes at some obscure point. To overcome the rotation, when present, hold each plate firmly against its ridge during side to side movements until, when released, no further tendency to rotate is noted. The grinding may then be completed by the patient unassisted.

Following the side to side grinding it is advisable to have the patient grind, for the incising articulation, by protruding and retruding the lower jaw till free motion can be made.

If the centrals strike during this operation and the upper cuspid and lower bicuspid do not, shorten the upper or lower centrals till the cuspids come into contact. If the cuspids interfere reverse the operation.

